# 1307. Vaso-occlusive Crisis is Associated with Subsequent Osteomyelitis in Pediatric Sickle Cell Disease

**DOI:** 10.1093/ofid/ofad500.1146

**Published:** 2023-11-27

**Authors:** Kate Shapiro, Joshua Wolf, Rohith Jesudas, Jeffrey C Winer, Sandra Arnold

**Affiliations:** St. Jude Children's Research Hospital, Memphis, Tennessee; St. Jude Children's Research Hospital, Memphis, Tennessee; SJCRH, Memphis, Tennessee; Le Bonheur Children's Hospital/UTHSC, MEMPHIS, Tennessee; University of Tennessee Health Science Center, Memphis, Tennessee

## Abstract

**Background:**

Children with sickle cell disease (SCD) commonly develop microvascular infarcts of bone known as vaso-occlusive crisis (VOC) and are also at high risk of osteomyelitis (OM). VOC and OM can be challenging to distinguish due to overlapping clinical features. VOC might predispose to OM, or an initial misdiagnosis of OM as VOC might lead to delayed diagnosis of OM. This study aimed to test whether VOC was associated with subsequent development of OM in children with SCD.

**Methods:**

A retrospective Pediatric Health Information System (PHIS) database study (2010-2021) was performed. ICD-code data related to common diagnoses in pediatric patients with SCD were extracted. The rate of VOC within 30 and 180 days preceding episodes of OM was compared to the rate preceding acute chest syndrome (ACS), another common diagnosis in children with SCD but unlikely to be anatomically related to OM. Odds ratios (OR) were used to evaluate the association between these diagnoses.

**Results:**

Of 22,818 PHIS participants with SCD, 13,699; 450; and 7,327 had ≥ 1 episode of VOC, OM and ACS, respectively. The distribution of OM diagnoses was similar across pre-specified age groups (0-4, 5-12, and 13-18 years). The odds ratio of VOC preceding a diagnosis of OM vs. ACS was 2.15 within 30 days (95% CI: 1.82-2.54; p= 3.68 x 10^-18^), and within 180 days was 1.39 (95% CI: 1.20-1.62; p=2.04 x 10^-5^).

VOC Preceding Osteomyelitis vs. Acute Chest Syndrome

**Figure:**
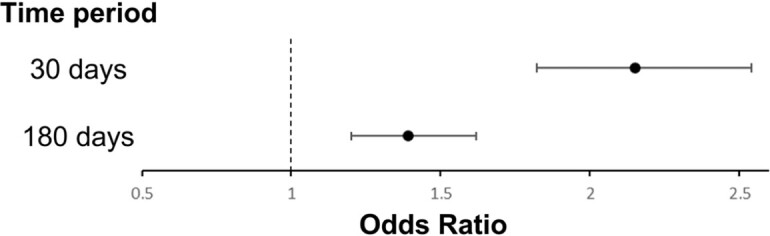

**Conclusion:**

VOC was significantly more common in the days preceding a diagnosis of OM than of ACS. The strength of association was stronger within 30 days prior to OM than 180 days. These results suggest possible misdiagnosis of OM since VOC might delay OM diagnosis in some cases, that recurrent VOC might be misdiagnosed as OM, or that VOC can directly contribute to risk of OM in some cases. Limitations of this study include possible incomplete adjustment for unmeasured variables which could confound the analysis. More research is needed to confirm and evaluate the mechanism behind this association. Understanding the relationship could lead to improved screening and treatment of both VOC and OM.

**Disclosures:**

**Joshua Wolf, MBBS, PhD**, Karius Inc.: Grant/Research Support|Merck Inc.: Participation in industry-sponsored research **Sandra Arnold, MD**, Contrafect: Grant/Research Support|Enanta: Grant/Research Support|Moderna: Grant/Research Support|Pfizer: Grant/Research Support

